# Molecular Effects of Irradiation (Cobalt-60) on the Control of *Panonychus citri* (Acari: Tetranychidae)

**DOI:** 10.3390/ijms161126004

**Published:** 2015-11-11

**Authors:** Ke Zhang, Lingyan Luo, Xieting Chen, Meiying Hu, Qiongbo Hu, Liang Gong, Qunfang Weng

**Affiliations:** 1 Key Laboratory of Pesticide and Chemical Biology, Ministry of Education of China, South China Agricultural University, Guangzhou 510642, China; wengweng@scau.edu.cn (K.Z.); luolingyan0915@gmail.com (L.L.); xtchan@outlook.com (X.C.); humy@scau.edu.cn (M.H.); hqbscau@126.com (Q.H.); 2 Key Laboratory of Plant Resource Conservation and Sustainable Utilization, South China Botanical Garden, Chinese Academy of Sciences, Guangzhou 510650, China

**Keywords:** *Panonychus citri*, gene expression profile, sterility exposure, radiation-induced, apoptosis pathway

## Abstract

The effective dose of irradiation to control pest mites in quarantine has been studied extensively, but the molecular mechanisms underlying the effects of the irradiation on mites are largely unknown. In this study, exposure to 400 Gy of γ rays had significant (*p* < 0.05) effects on the adult survival, fecundity and egg viability of *Panonychus citri*. The irradiation caused the degradation of the DNA of *P. citri* adults and damaged the plasma membrane system of the egg, which led to condensed nucleoli and gathered yolk. Additionally, the transcriptomes and gene expression profiles between irradiated and non-irradiated mites were compared, and three digital gene expression libraries were assembled and analyzed. The differentially expressed genes were putatively involved in apoptosis, cell death and the cell cycle. Finally, the expression profiles of some related genes were studied using quantitative real-time PCR. Our study provides valuable information on the changes in the transcriptome of irradiated *P. citri*, which will facilitate a better understanding of the molecular mechanisms that cause the sterility induced by irradiation.

## 1. Introduction

The citrus red mite, *Panonychus citri* (Acari: Tetranychidae), is a cosmopolitan agricultural pest with over 80 host plants, including hosts such as citrus, almond and rose [1,2,3]. Additionally, as a serious allergen, *P. citri* contributes to the development of rhinitis and/or asthma in citrus farmers [4]. The control of *P. citri* depends primarily on chemical pesticides, but the excessive use of hexythiazox against *P. citri* increased the resistance to this acaricide 3532-fold [3]. As an alternative means of control, irradiation was used as a quarantine treatment to control the mites. For example, the dose required for lychee to be cleaned of mites is 350 Gray [5]. Additionally, our previous study demonstrated that low doses of irradiation increased the level of oxidative stress in *P. citri* [6]. However, little is known about the effects of high doses of irradiation on molecular and physiological function in *P. citri*.

In the early 1970s, scientists conducted studies using radiation against mites to obtain genetic information on their androgenesis as well as the cytological effects of and the genetic damage caused by radiation [7,8,9]. Recently, exposing a model insect, *Drosophila*, to high-dose irradiation induced arrest of the cell cycle, apoptosis, and developmental defects during oogenesis [10]. Ionizing radiation causes double-stranded breaks in DNA, which result in pathological cellular responses, such as arrest of the cell cycle, DNA repair and apoptosis [11]. Moreover, γ irradiation against the *Taenia solium* metacestode caused apoptosis, which led to the elucidation of the mechanisms underlying γ irradiation inhibiting the normal development of the *T. solium* metacestode in the adult worm [12]. In addition, the cause of female infertility from irradiation was confirmed to be a mutation of *Chk2*, a gene that is indispensable for fertility and viability [13]. In most cases, the radiation for mutants was conducted randomly, and information on the mechanisms for sterility after radiation treatment is limited. Therefore, it is both urgent and necessary to have a comprehensive understanding of the effects of radiation treatment on the differential expression of genes.

The goal of this study is to investigate the possible molecular mechanisms about effects of high doses of radiation on *P. citri*, and hence we described the transcriptome of mites exposed to radiation using the Illumina Hiseq™ 2000 platform. To identify the genes that might change in response to radiation between sterile and healthy mites, we performed a DGE (Digital Gene Expression) analysis in normal adults and compared this analysis with two different time points after the irradiation. This approach was a shortcut to identify the genes involved in the irradiation-induced sterility in *P. citri*. Our results indicate that apoptosis is involved in the radiation-induced negative regulation of generation, which leads to infertility in *P. citri*.

## 2. Results and Discussion

### 2.1. Effects of γ Irradiation on Mortality and Reproduction of Adult Mites

The goal of irradiation is not to cause severe mortality, but to prevent development or reproduction because 100% acute mortality required very high doses of irradiation that could not be tolerated by most fresh commodities [14]. In the present study, with the results shown in Table 1, the mortality increased significantly (*p* < 0.05) after the irradiation treatment from day 1 to day 9, and the number of eggs laid by the irradiated adults decreased significantly; furthermore, these eggs did not hatch to produce the next generation. Consistent with our data, Castro *et al.* (2004) reported that the mortality of *Brevipalpus chilensis* increased to 46.6% when the irradiation dose was increased to 350 Gy; additionally, a decrease in the capacity for oviposition occurred when the irradiation dose was increased to 300 Gy, and *B. chilensis* was not viable [15]. Moreover, our previous research suggested that irradiation with 400 Gy from Co^60^γ was an effective postharvest technique to mitigate the risk of *P. citri* on citrus fruit [6]. We investigated the possibility of using irradiation as a quarantine treatment for *P. citri*; because of parthenogenesis in *P. citri* females, a suitable dose of radiation without effects on the citrus fruit was measured by F_1_ sterility.

**Table 1:** F_1_ fecundity (no. of eggs), percent of egg hatch and mortality of adult *P. citri* irradiated with Co^60^γ-rays.

Treatment Dose (Gy)	0	1	2	5	7	9	14	Mean No. of Eggs	F_1_ Hatched Eggs
0	0.67a	2.67a	6.00a	10.00a	14.00a	46.67a	92.67a	1133	94.6
400	2.00a	12.00b	22.67b	42.67b	64.67b	84.00b	96.67a	375	0

Means within in each column followed by the same letter are not significantly different (*p* < 0.05).

### 2.2. Effects of γ Irradiation on Adult DNA and Egg Development

**Figure 1:**
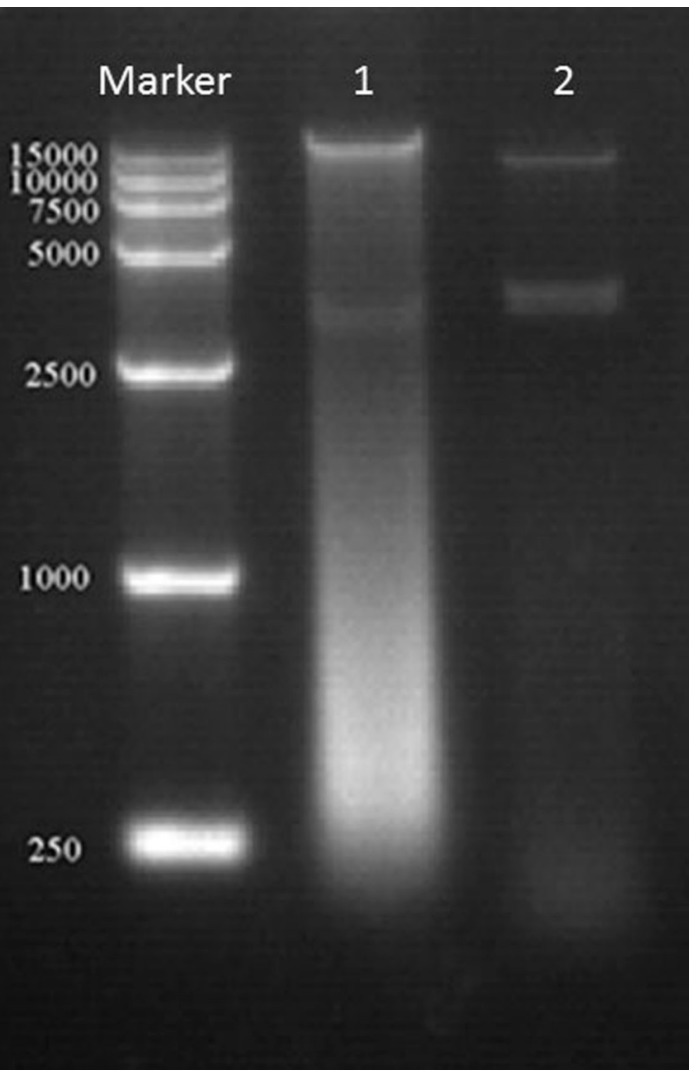
DNA fragmentation was examined with agarose gel electrophoresis. 1 represents the irradiated mites, and 2 represents the normal mites.

The fragmentation of DNA is a common phenomenon with apoptotic cell death, which is conserved in different species, including *Drosophila melanogaster* and *Bombyx mori* [16,17]. The results of the DNA fragmentation, as determined by agarose gel electrophoresis, are illustrated in Figure 1. A significant fragmentation of DNA was observed in the samples irradiated with more than 400 Gy of γ rays, and the DNA fragments, which varied in size between 250 and 2500 base pairs, were clearly visible after the electrophoresis. Apoptosis is well known to be an orderly cellular process of suicide in response to various stimuli [18]. It has been reported that ionizing radiation could cause cellular response in relation to the genes that mediated the complex regulatory pathways including cell cycle, apoptosis, and DNA repair [19]. Irradiation causes double-stranded breaks in the DNA, which results in three well-studied cellular responses: the regulation of the cell cycle by checkpoints, DNA repair and apoptosis [20]. Our results showed that the 400 Gy of γ rays caused serious damage to the chromosomes of *P. citri* and that the chromosome fragments might lead to delays in cell division or may even cause some cells to stop dividing or die.

**Figure 2:**
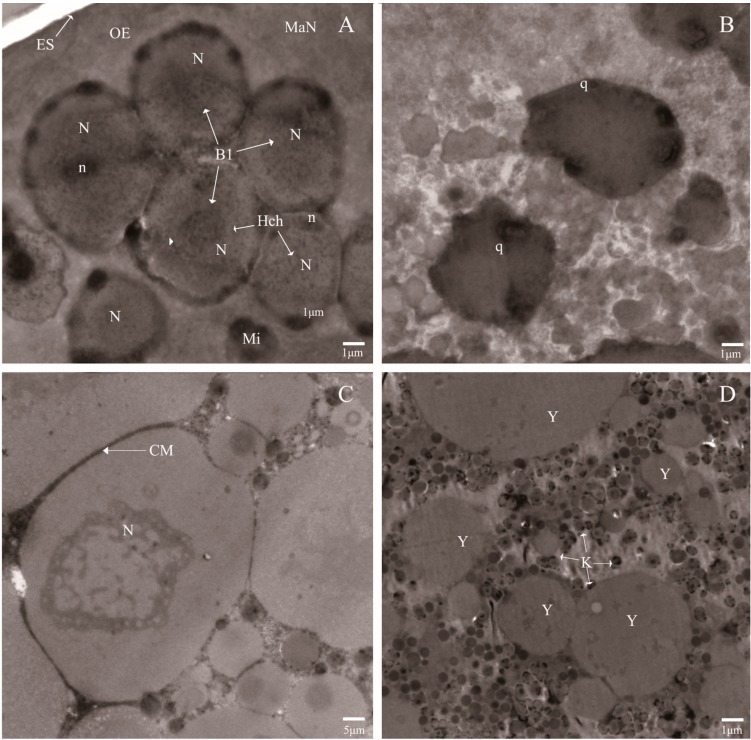
Ultra-structural features of the eggs laid by the irradiated mites (**B**,**D**) and the normal mites (**A**,**C**). Blastomeres (Bl); Nuclei (N); Outer envelope (OE); Micromere (Mi); Egg shell (ES); Nucleolus (n); macromere (MaN); Cell membrane (CM); Apoptotic body (q); Heterochromatin islands (Hch); Degenerating nucleoli (k); and Gathered yolk (Y).

Compared with the normal eggs, the ultra-structural investigation showed that variations occurred in the eggs laid by the irradiated adults. As shown in Figure 2B,D, the plasma membrane was destroyed, which resulted in cytoplasmic leakage and the overflowed cytoplasm forming the gathered yolk, and the nuclei contain compact granular masses, which may be formed by chromatin associated with the nuclear envelope. However, the normal development of eggs were shown well-organized morphology and cell membrane system. Moreover, the macromere (MaN) was found at the position of newly formed OE (outer envelope), and the other blastomere nuclei well aligned with the micromere (Mi) and possessed different particle sizes (Figure 2A,C). It was reported that high-dose irradiation easily causes complete egg sterility [21]. Our data were consistent with this observation and demonstrated that high-dose irradiation damaged the endomembrane system of the eggs of *P. citri*, particularly the plasma membrane, which led to the condensed nucleoli and the gathered yolk.

Apoptosis is the process of programmed cell death that depends on the expression of many gene products, such as caspases [22]. Although this study did not examine the characteristics of apoptosis in preimplantation embryos, the traits that were exhibited were fully along with the process of programmed cell death. A previous study confirmed that some types of cell deaths were found in the preimplantation embryos, particularly during the postcompaction stages, and some types of blastomere DNA fragmentation and embryonic cell cycle arrest were agreed with an autophagic mode of cell death; thus, the regulation of this phenomenon by a subset of genetic and biochemical events that controlled apoptosis was supported by this study [23].

### 2.3. An Overview of the Transcriptome of P. citri

The total number of the Illumina sequencing short reads was 78,795,170 (Table 2, NCBI SRA No. SRX800412), and 60,619 contigs were assembled with a mean length of 636 bp. 54,595 unigenes were further assembled by using the paired-end reads and Trinity, with a mean length of 604 bp. The assembly generated a large number of contigs, including 10,708 contigs were >1000 bp, and 8644 unigenes were >1000 bp. Of note, the assembly and annotation of different *P. citri* cDNA libraries resulted in different data outputs. For example, a report on the transcriptomes of resistant and susceptible strains of *P. citri* found that the number of unigenes was 34,159 and 30,466 and that the mean length of the unigenes was 489 and 536 bp, respectively [3]. All of the assembled and annotated contigs were deposited in the NCBI with the BioProject ID: SUB773748.

**Table 2:** Summary for *P. citri* transciptome.

	Total Number	Total Nucleotides (nt)	Average Length (bp)	N50
Reads	78,795,170	7,879,517,000	-	-
Contigs	60,619	38,562,819	636	932
Unigenes	54,595	33,003,960	604	868

Based on searching the unigenes using BLASTx against Nr, Swiss-Port, COG and KEGG, 29,904 unigenes were annotated with a ratio of 54.77% in all the unigenes. Furthermore, the unigene sequences were evaluated by the Clusters of Orthologous Groups (COG) classification. Of the 28,872 Nr hits, 11,356 sequences were shown a COG classification (Figure S1). Among the 25 COG categories, the cluster for “General function prediction only” (3695; 32.54%) was the largest group, followed by “Carbohydrate transport and metabolism” (2134; 18.79%), “Transcription” (1931; 17.00%), “Translation, ribosomal structure and biogenesis” (1832; 16.13%), and “Posttranslational modification, protein turnover, chaperones” (1630; 14.35%). The categories “extracellular structures” (25; 0.22%) and “nuclear structure” (13; 0.11%) had the fewest corresponding genes. Apoptosis is generally considered as a process of programmed cell death, which associates with the environmentally determined elimination of cells [24]. In this study, the sequences associated with pathways that could be identified are presented in Table 3. The cell cycle and apoptosis play important roles in the genetic networks that explain the radiation induced sterility, such as the p53 signaling pathway, which was investigated with 84 unigenes in relation to the high-dose radiation on *P. citri* (Table 3 and Table S1). As reported, the p53 pathway played a critical role in regulating the response to DNA damage, which led to micronucleus formation in chronically irradiated cells [25].

**Table 3:** Selected pathways are enriched and involved in the pathways of apoptosis, cell death and the cell cycle induced by the irradiation of *P. citri*.

Pathway	All Genes with Pathway	Pathway ID
	Annotation (16,065)	
P53 signaling pathway	84 (0.52%)	ko04115
Apoptosis	108 (0.67%)	ko04210
Regulation of autophagy	39 (0.24%)	ko04140
MAPK signaling pathway	434 (2.7%)	ko04010
Calcium signaling pathway	296 (1.84%)	ko04020
Protein processing in endoplasmic reticulum	488 (3.04%)	ko04141
Lysosome	474 (2.95%)	ko04142
Cell cycle	296 (1.84%)	ko04110
Wnt signaling pathway	158 (1.61%)	ko04310
Chemokine signaling pathway	243 (1.51%)	ko04062
Neurotrophin signaling pathway	222 (1.38%)	ko03015

**Figure 3:**
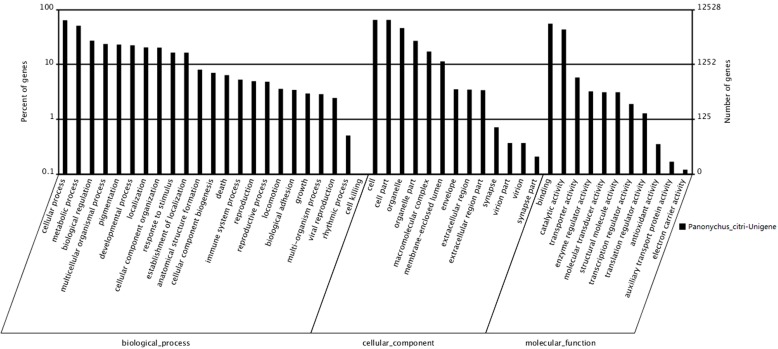
Gene Ontology classifications of assembled unigenes. A total of 12,528 unigenes were categorized into three main categories: biological process, cellular component and molecular function.

When the unigenes were blasted against the NCBI nonredundant (nr) database using BLASTX with a cutoff e-value of 10^−5^. Of the 54,595 unigenes, 28,872 (52.9%) returned at least one match with the *e*-value >10^−5^. As there is lack of genome and EST information for *P. citri*, 47.1% of the unigenes could not be matched to any known genes in the Nr (Non-redundant) database (Available online: http://blast.ncbi.nlm.nih.gov/Blast.cgi). According to sequence homology, 12,528 unigenes were categorized into 47 Gene Ontology (GO) terms (Figure 3). The results showed that among the three categories, the terms cellular progress and metabolic progress occurred most frequently in the ontology of biological processes, whereas the terms cell, cell part and organelle and the terms binding and catalytic activity occurred most frequently in the ontology of cellular components and molecular functions, respectively. The response to stimulus, death, reproduction and viral reproduction were highly represented groups in the biological process category. In the “cellular process” category, 8015 unigenes were annotated, which suggested that our study identified novel genes involved in the cell cycle. The cell and organelle parts were the dominant groups in the “cellular component function” category. Though a high percentage of the genes in the “molecular function” category were from the “binding” (55.27%) and the “catalytic activity” (43.37%) groups. In the “cell killing” groups, only a few (10; 0.08%) unigenes were annotated, which suggested that the 400 Gy of irradiation caused damage to the cell before death (Table 3). Compared with a similar study on the *P. citri* transcriptome, the number of unigenes annotated against nr, KEGG, COG and GO were 32,535, 22,725, 15,412 and 10,421, respectively in which the COG annotations were involved in 25 molecular families, including such as general function prediction, translation, ribosomal structure and biogenesis [23].

### 2.4. Profiles of Transcript Abundance through Time Intervals

To understand the dynamic biological process of transcript abundance at two and five days after the irradiation treatment, the expression patterns of all differentially displayed genes were further analyzed using the expression level of the patterns considered as a common reference. According to this analysis, the 4986 differentially expressed genes were grouped into eight profiles using STEM (Short Time-series Expression Miner).

**Figure 4:**
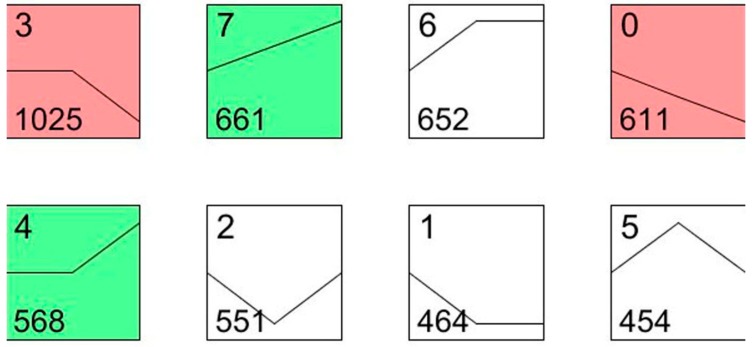
STEM (Short Time-series Expression Miner) clusters of expression profiles. The profiles are ordered based on the *p*-value significance of the number (at bottom-left corner) of genes assigned *versus* expected. The colored square frame denotes significant profiles (*p*-value ≤ 0.01). Each graph displays the mean pattern of expression (black lines) of the profile genes. The number of profiles in each cluster is at the top left corner of each STEM and the number of genes in the bottom left corner of the panels. The *x*-axis represents stages, and the *y*-axis represents log2-fold change in gene expression.

As shown in Figure 4, profile 0 and profile 7 showed a monotonic decrease and increase, respectively, among the three time intervals. The genes that coded for chitinase (Unigene002740), cytochrome P450 (Unigene0012520) and ubiquitin hydrolase (Unigene0020738) were included in profile 0, and the gene that coded for the apoptosis regulator BAX (Unigene0053037) was included in profile 7. The most represented pattern was a mass increase after the irradiation from day 2 to day 5 (1025 genes, profile 3), which included the transcripts involved in ubiquinol-cytochrome c (Unigene0015549), p38 MAP kinase (Unigene0013647), and cytochrome P450 (Unigene0026480). The transcripts within profile 4 presented a curve in which the expression of 568 genes increased from day 2 to day 5, including caspase 7 (Unigene0023555) and autophagy-related protein 4 (Unigene0024949). Most of these genes are involved in apoptosis, cell death and the cell cycle. For example, caspase 7 was reported to be involved in the cleavage of claspin during apoptosis, which inhibited the Chk1 pathway [26]. Cytochrome P450 is a large family of genes in insects, which plays important roles in insecticide resistance and hormone metabolism. In our study, these P450 genes, such as Unigene0026480 and Unigene0012520, were expected to be involved in the *P. citri* defense against the environmental stress caused by the irradiation treatment.

### 2.5. Apoptosis Pathway Assignment by KEGG

Apoptosis is a type of programmed cell death, and many cells undergo such a programmed cell death during normal development or aging via a kind of homeostatic mechanism to keep cell populations in tissues. Apoptosis also serves as a defense responses in relation to the immune mechanism while cells were damaged by disease or noxious agents. It is not all cells have to die in response to multitudinous physiological and pathological stimulus. For example, irradiation or drugs used for pest control caused DNA damage in some cells, which led to apoptotic death through the MAPK signaling pathways or a p53-dependent pathway [27].

In our study, 54,595 unigenes were annotated by KEGG, in which 23,092 unigenes were assigned to 275 KEGG pathways. The MAPK signaling pathways had the most representation among the unigenes (434 members, 2.7%), which was followed by apoptosis (108 members, 0.67%). These identified pathways provide a valuable resource to investigate specific genes, functional annotations and pathways for research on *P. citri* damaged by irradiation. Notably, 1397 unigenes involved in apoptosis were confirmed including 11 pathways (Table 3). The p53 is an important factor in the control of insect physiology and an important part of the apoptosis pathway [27]. The induction of p53 occurs in response to a range of genotoxic or nongenotoxic stresses, which result in the biological outcomes of arrested growth or apoptosis. In the present study, the p53 signaling pathway was found in the KEGG pathway that involved 84 (0.52%) unigenes. Details of the p53 signaling pathway are shown in Figure S2. This pathway will be given promising for further studies on the effects of irradiation-related processes on the pathway leading to sterility in *P. citri*.

**Figure 5:**
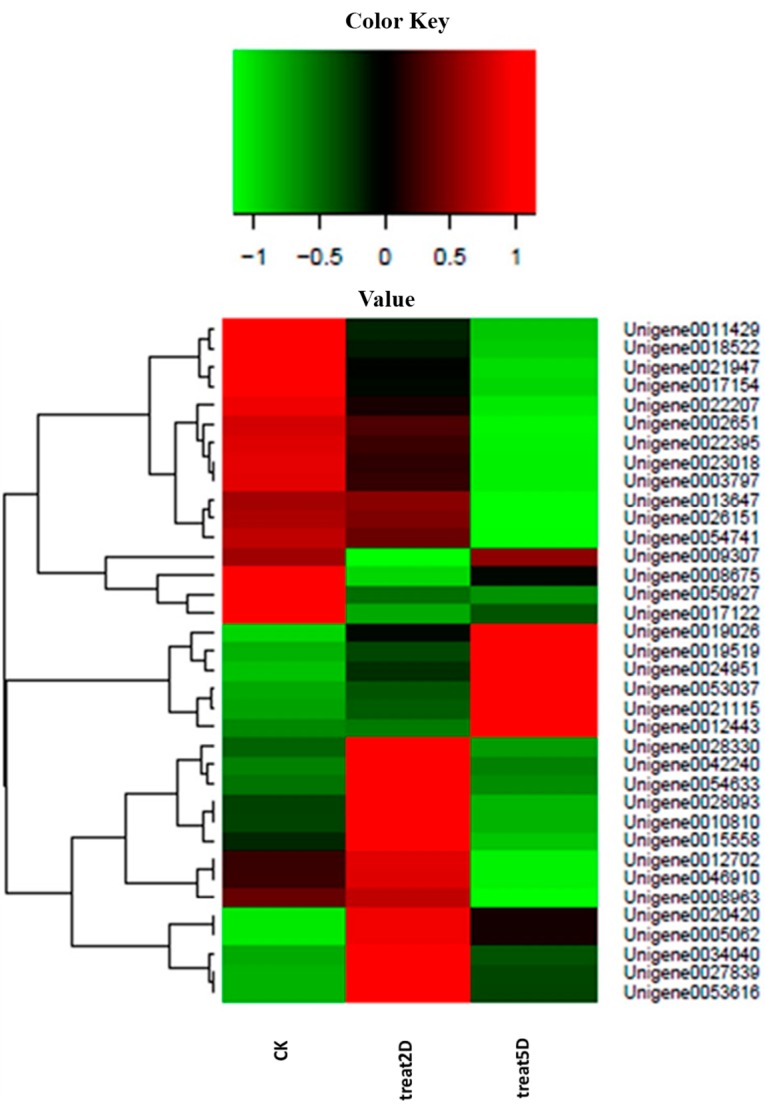
Differential expression of genes related with terms in the *P. citri* STEM. Each column represents an experimental sample (e.g., CK, 2D and 5D), and each row represents a gene. Differences in expression are shown in different colors. Red indicates high expression, and green indicates low expression.

**Figure 6:**
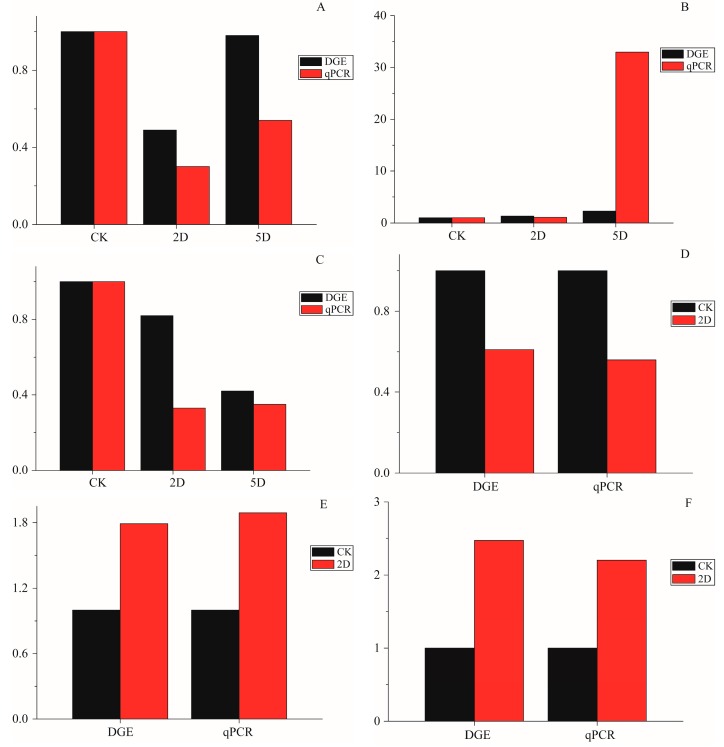

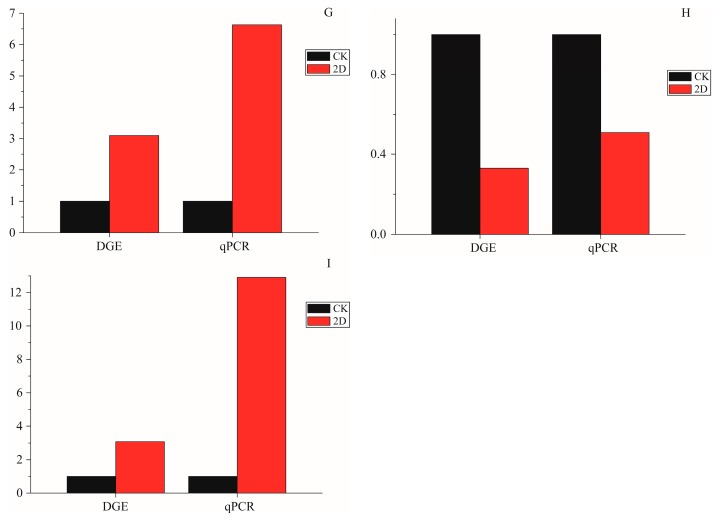
The qPCR data for the expression pattern validation of selected genes. The x-axis indicates treatment, and the y-axis indicates relative expression level. The following selection of genes was tested, with their descriptions. (**A**) Apolipoprotein D (Unigene0009307); (**B**) Calmodulin-like (Unigene0019519); (**C**) Breakpoint cluster region protein (Unigene0022395); (**D**) Serine protease (Unigene0017154); (**E**) Intestinal-type alkaline phosphatase 1 (Unigene0015558); (**F**) Cu/Zn superoxide dismutase (Unigene0054633); (**G**) Microtubule-associated proteins (Unigene0021115); (**H**) DNA repair endonuclease XPF-like (Unigene0022207); and (**I**) autophagy-related 4 (Unigene0024951).

To improve our knowledge on the gene expression obtained from the *P. citri* transcriptome, three DEG libraries were created to analyze the gene expression profiles; a large amount of genes showed significantly changed expression and involvement in the radiation-induced sterility. These genes were related to catalytic activity, autophagy, cellular response to stimuli, oxidoreductase activity, embryo development, hydrolase activity, and signal transduction. Furthermore, these genes had significantly different expression patterns among the three time intervals (Figure 5). The assumption was that these genes might be induced independently of a p53 pathway at day 2 after irradiation, but the levels of expression increased at day 5, which might contribute to the delayed apoptotic response. For example, Unigene0042240 showed higher expression levels day 2 after irradiation compared with the control group and day 5 after irradiation; this result was homologous to MDM2 because an E3 ubiquitin ligase directed p53 ubiquitylation and degradation [28]. Additionally, Unigene0020420 and Unigene0053037 showed higher levels of expression at day 2 and day 5 than in the CK, which were functional for stimuli (Unigene0053037 was homologous to Bax, which was activated in response to radiation) [29]. Other genes with significantly different levels of expression in the three time intervals were also annotated, such as Laminin subunit α (Unigene0026151), hydrolase activity (Unigene0008675 and 0017122), catalytic activity (Unigene0017154, 0003797, and -0054633), oxidoreductase activity (Unigene0012443 and 0019026), and autophagy (Unigene0021115 and 0024951). To verify a subset of the DGE data, the qRT-PCR analyses were performed. The expression of 9 genes that were annotated in apoptosis-related pathways were studied using qRT-PCR. The results showed a similar direction of expression induced by irradiation between the DGE and the qRT-PCR results (Figure 6), and the qRT-PCR analysis confirmed the same tendency of expression detected by the DGE analysis.

Until now, in sterile *P. citri*, no study examined which genetic program was affected that regulated a vital process, such as cell division, differentiation or apoptosis. This study provides the first evidence that apoptosis operates and is induced by γ radiation in *P. citri*. Although the detailed functions of genes in relation to the genetic pathways of sterility in *P. citri* remain unknown, the current transcriptome analysis provides valuable gene sequence information on *P. citri* sterility, which may facilitate further investigations into the details of sterility caused by irradiation.

## 3. Experimental Section

### 3.1. Mite Samples and Bioassay

A colony of citrus red mites, *P. citri*, was collected from citrus plants in a citrus orchard (South China Agricultural University, Guangzhou, China; 113.34′E, 23.16′N); pesticides were not applied for many years in the orchard. For the present study, the mites were reared in incubators at 26 ± 1 °C, 70%–80% relative humidity, and a 16:8 h (light:dark) photoperiod for several generations. The mites were irradiated with Co^60^γ-rays at the Furui high-energy Technology Co. Ltd. (Guangzhou, China).

Freshly picked citrus leaves were washed with water, the leaf edge and petioles were wrapped with wet absorbent cotton, and the leaves were placed in a 12-cm-diameter petri dish. A wet sponge was placed on the petri dish bottom to keep the leaves moist. The female adult mites were selected independently to a consistent size and were irradiated with 400 Gy of γ rays. The mites were placed to feed on the previously prepared citrus tree leaves, each blade of which had 50 mites, and the mites remained on the leaves for 12 h for spawning. The assays were conducted in quadruplicate. The normal female adult mites without irradiation were used as negative controls. After the 12 h, the adult mites were gently removed with a brush, and the number of eggs per leaf was recorded. After irradiation, the leaves were maintained in separate plastic boxes and incubated under the conditions described above. To determine whether the eggs that were laid by irradiated females hatched, the development of the mite population (eggs, larvae, nymphs, and female and male adults) was assessed by visual observation. The evaluations were performed every other day until 14 days after the irradiation and were then performed weekly until 60 days after the treatment. The mites were removed from the citrus leaf and frozen immediately in liquid nitrogen for RNA extraction.

### 3.2. DNA Detection and Transmission Electron Microscopy (TEM) Observation

The total DNA was extracted from two hundred mites two days after the irradiation treatment with the TIANamp Genomic DNA Kit (Tiangen Biotech, Co., Beijing, China). The DNA of the normal mites was extracted as the control set. The concentration and quality of the DNA were verified using a 2100 Bioanalyzer (Agilent Technologies, Santa Clara, CA, USA), and the results then checked with 0.8% agarose gel electrophoresis.

For the TEM examinations, the eggs laid by irradiation treated and un-treated female adults, were collected and embedded in 4% agar, which then were transferred to 2.5% glutaldehyde to fix for more than 4 h at 4 °C. The fixed specimens were washed with 0.1 M sodium-phosphate buffer (PB) and postfixed in 1% OsO_4_ for 2 h. Then, the samples were washed with 0.1 M PB and dehydrated with a concentration gradient of ethanol solutions (30%, 50%, 70%, 80%, 90%, 95%, and 100%). Finally, the samples were embedded in Epon and cut into serial semi-thin sections (2 mm). After double staining with uranyl acetate and lead citrate, the semi-thin sections were examined with a transmission electron microscope (JEM-1010, JEOL, Tokyo, Japan) at an accelerating voltage of 100 kV.

### 3.3. Construction of the Mite cDNA

The cDNA library was generated to obtain an overview of the *P. citri* transcriptome, by using SMART cDNA Library Construction Kit (Takara, Tokyo, Japan) from an equal mixture of adult mite RNA isolated from the control mites and from the mites 2 and 5 days post irradiation. Two biological replicates were performed for each sample, which were obtained from two independent irradiation treated and un-treated *P. citri*. The poly (A) mRNAs were isolated using oligo-dT beads (Thermo Fisher, MA, USA). All mRNAs were broken into short fragments (200 nt) via the addition of fragmentation buffer. The first-strand cDNA was generated using random hexamer-primed reverse transcription and was followed by the synthesis of the second-strand cDNA using RNase H and DNA polymerase I. The QIAquick PCR extraction kit was used to purify the cDNA fragments which were then washed by EB buffer for end reparation poly (A) addition and ligation to sequencing adapters. Agarose gel electrophoresis was used to separate the cDNA, which was than extracted from the gels, the cDNA fragments (200 ± 25 bp) were purified and enriched using PCR for final construction of the cDNA library. The cDNA library was sequenced by the next-generation sequencing platforms (Illumina HiSeq™ 2000, Salt Lake City, UT, USA) using the single-end paired-end technology in a single run. The original data were treated using the Illumina GA Pipeline (version 1.6; Illumina Inc.) and the paired-end reads with 100 bp in length were obtained [30]. By removing the low-quality sequences using the perl program (Available online: https://www.perl.org/), the clean reads were assembled by Trinity in order to construct unigenes.

### 3.4. Bioinformatics Analysis

The filtered transcripts were annotated with BLASTx, a tool developed to evaluate the similarity between two sequences, against the NCBI nonredundant protein database (Nr) (Available online: http://www.ncbi.nlm.nih.gov/), the Clusters of Orthologous Groups of proteins database (COG) (Available online: http://www.ncbi.nlm.nih.gov/COG/), and the KEGG and the Swiss-Prot (Available online: http://www.expasy.ch/sprot), with an e-value cutoff of <10^−5^ [31]. The protein sequence in the database that had the highest similarity score was used to obtain the functional annotation result of the related Unigene. The Unigenes were annotated by the Blast2GO to obtain the results for Gene Ontology (GO) (Available online: http://www.geneontology.org/), which is a database that contains a series of terms that describe gene information using three ontologies: molecular function, biological process and cellular compound [32]. WEGO is a statistical tool, which was chosen to analyze the annotation results of the unigenes in the GO database (Available online: http://wego.genomics.org.cn/cgibin/wego/index.pl). Using the short time series expression miner (STEM) V1.3.8 (Available online: http://www.cs.cmu.edu/~jernst/stem/), the gene expression profile was studied in relation to dynamic biological processes [33].

### 3.5. Quantitative-RT-PCR

To confirm these results, an aliquot of the same RNA samples was used for the Q-RT-PCR analysis. The 3.0 μL samples of RNA were reverse transcribed into cDNA with the PrimeScript^TM^ RT reagent Kit with gDNA Eraser (Perfect Real Time). As shown in Table S1, primer premier 5.0 designed the primers. The PCR used SYBR^®^ Premix Ex TaqTM II, 2.0 μL of cDNA, and 2.0 μL of primers to set up and read in a BioRad iQ5 real-time PCR detection system (Bio-Rad, Hercules, CA, USA). The relative expression of the cDNA between irradiation treated and un-treated samples were calculated by first normalizing the endogenous reference gene (ELF1A; GenBank accession number: HM582444) and then normalizing the expression level in the untreated samples based on the 2^−∆∆*C*t^ method [34]. The Sigma Plot 12.0 software (Systat Software, Inc., San Jose, CA, USA) was used to perform the statistical analysis based on *t*-tests, with *p* < 0.05 indicating significance.

### 3.6. Statistical Analyses

A statistical analysis was performed to measure the possible significant difference of each treatment, and the means were separated with Duncan Multiple Range tests using the SPSS software (version 18.0, SPSS, Inc., Chicago, IL, USA), with analysis of variance (ANOVA). All statistical tests were significant at a level of 5%.

## 4. Conclusions

We propose that apoptosis plays a definitive and different role in the sterility pathway and in the various other biological pathways in *P. citri*. Thus, this type of programmed cell death might be a cellular mechanism to explain sterility in mites following irradiation. In our study, the overview of *P. citri* transcriptome after the treatment with irradiation has been provided according to the Illumina sequencing technology, which identified a large number of candidate genes associated with apoptosis, cell death and the cell cycle. These data could be used as valuable resource to investigate the sterility pathway and various other biological pathways in *P. citri*. Additionally, our study will be beneficial for the fundamental cellular processes responsible for enhanced tolerance to irradiation, which may allow some eukaryotes to better persist into the future in an era of life affecting radiation.

## Supplementary Materials

Supplementary materials can be found at http://www.mdpi.com/1422-0067/16/11/26004/s1.
