# Supplementary material for: Molecular Effects of Irradiation (Cobalt-60) on the Control of Panonychus citri (Acari: Tetranychidae)
Source: Int J Mol Sci. 2015 Nov 11;16(11):26964–77. doi: 10.3390/ijms161126004 (PMC4661862; doi:10.3390/ijms161126004)
Supplement: Supplementary file 1 [file ijms-16-26004-s001.pdf]

Supplementary Materials: Molecular Effects of Irradiation (Cobalt-60) on the Control of *Panonychus citri* (Acari: Tetranychidae)

Ke Zhang, Lingyan Luo, Xieting Chen, Meiying Hu, Qiongbo Hu, Liang Gong and Qunfang Weng

Table S1. Primers were used for the qPCR.

Gene ID	Protein	qPCR Primers (5′ to 3′)
Unigene0009307	apolipoprotein D	F: TGGTCAACGAGCTACCGAAT<br>R: GTCGTTGAGTTGTATCGGCA
Unigene0019519	calmodulin-like	F: TGAGGTTGATGCCGATGGTA<br>R: ACATGTCTCAATTCAGCAGCA
Unigene0022395	Breakpoint cluster region protein	F: TCCAAGCCAAACAAAACCCT<br>R: AGGGAATCTCATGGTGCTCA
Unigene0017154	serine protease	F: CAGGGCATTACTGTGGAGGA<br>R: AGCGAGTCTGTTCAGTTCCA
Unigene0015558	Intestinal-type alkaline phosphatase 1	F: CAAAGAGTCGCACTGGTACA<br>R: GTTTCCGTTTCCTGGTCCAC
Unigene0054633	Cu/Zn superoxide dismutase	F: AGGAAACATTGAAGCCGATCA<br>R: GTCCACCAGCATTTCCTGTG
Unigene0021115	microtubule-associated proteins 1A/1B light chain 3A-like	F: TTGTACCCCAACACCTTCCA<br>R: ATTCCCACCAGCGAGAAGAA
Unigene0022207	DNA repair endonuclease XPF-like	F: ACCATCGCCACACTTTTCAG<br>R: TGAGGTCAATAAGGTCAGCGA
Unigene0024951	autophagy-related 4	F: GGTTGTATGCTACGCTGTGG<br>R: TACCTTCAGTCGCACCCATT

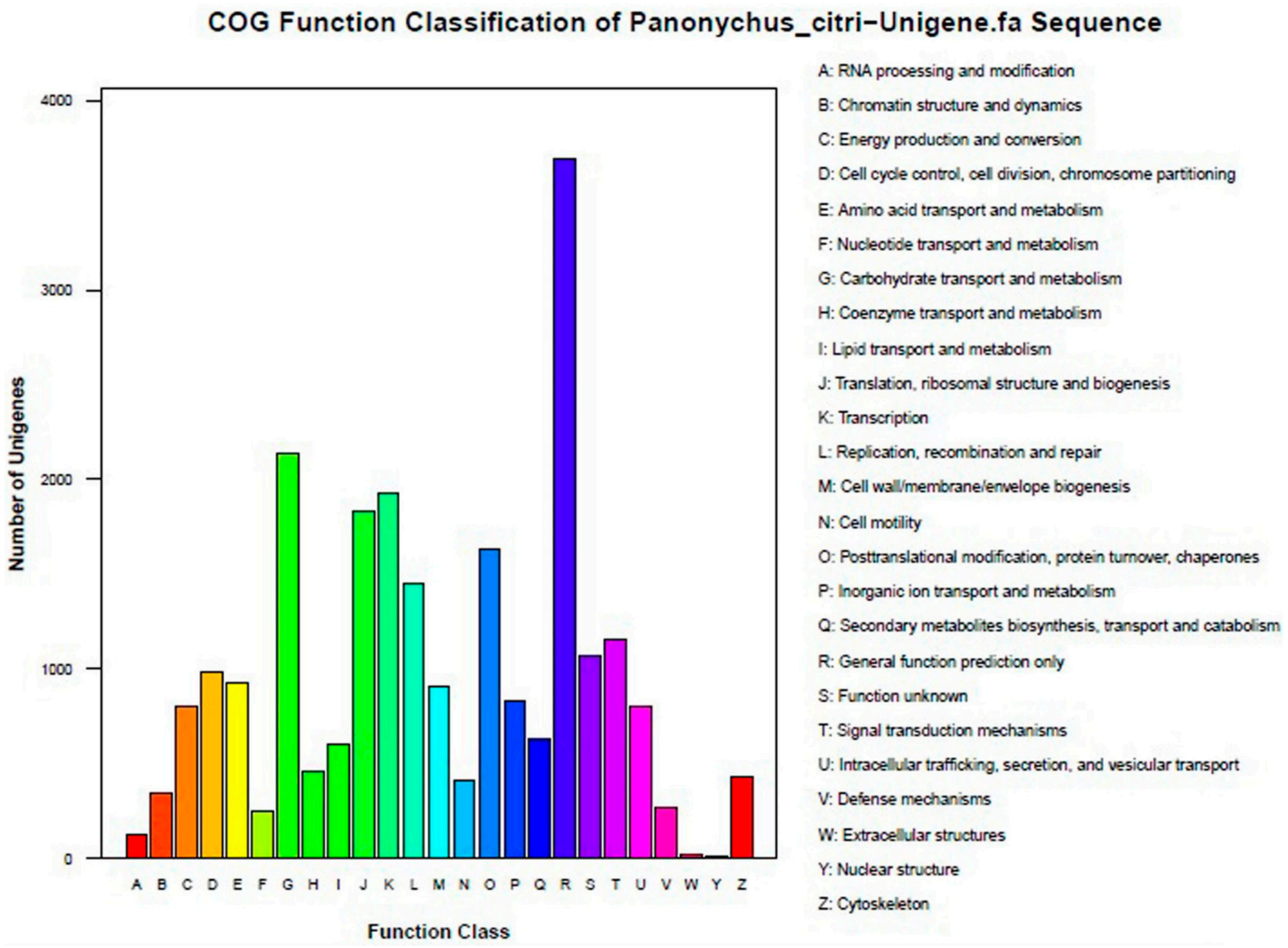

Figure S1. COG (Clusters of Orthologous Groups) function classification annotation of unigenes from *P. citri* transcriptome.

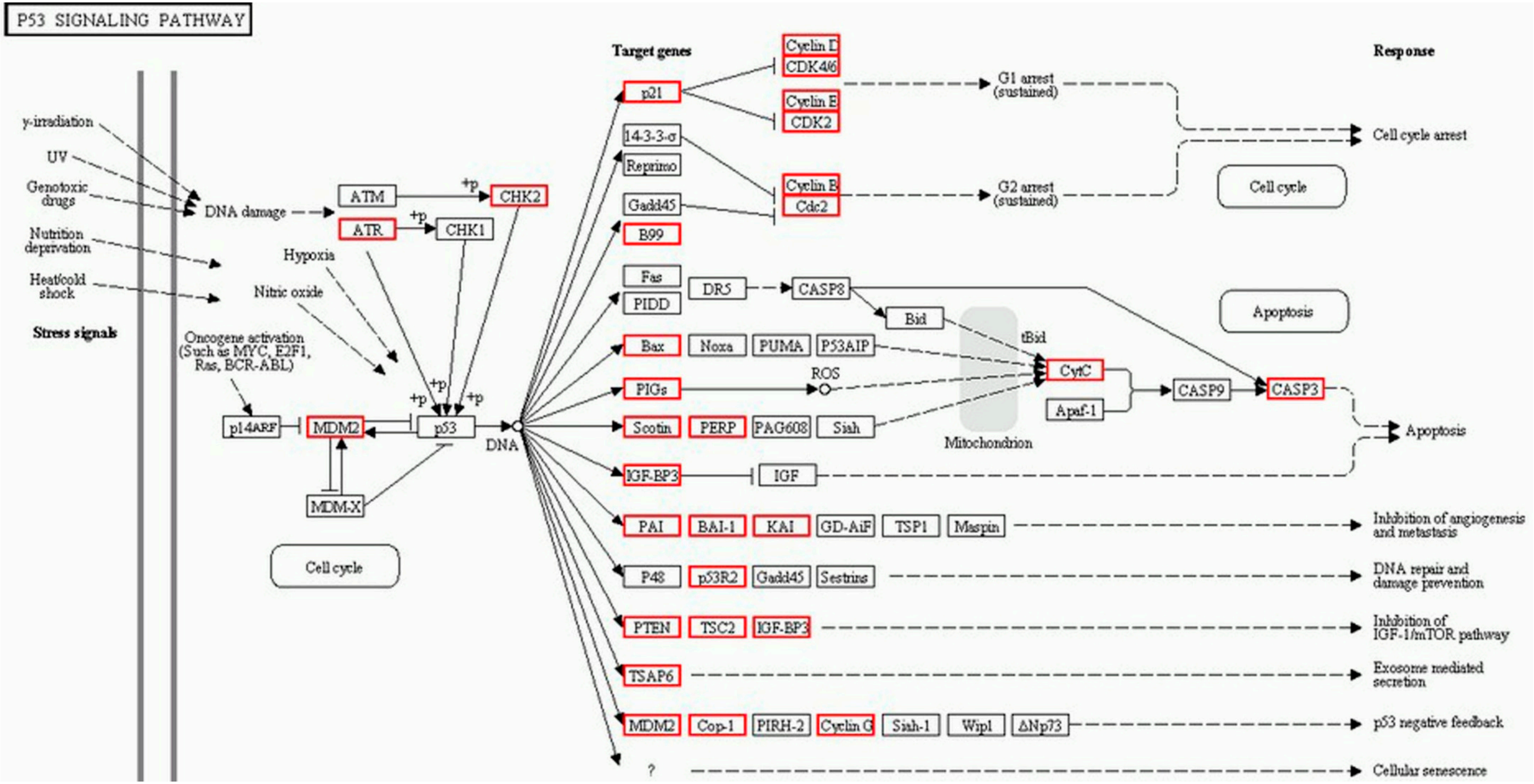

Figure S2. P53 signaling pathway for unigene annotated by KEGG. The unigenes with significant different expression are marked in red box. More information can refer to KEGG pathway: map04115 (Available online: http://www.genome.jp/dbget-bin/www_bget?pathway+map04115).
